# Combinatorial RNA Interference Therapy Prevents Selection of Pre-existing HBV Variants in Human Liver Chimeric Mice

**DOI:** 10.1038/srep15259

**Published:** 2015-10-20

**Authors:** Yao-Ming Shih, Cheng-Pu Sun, Hui-Hsien Chou, Tzu-Hui Wu, Chun-Chi Chen, Ping-Yi Wu, Yu-Chen Enya Chen, Karl-Dimiter Bissig, Mi-Hua Tao

**Affiliations:** 1 Graduate Institute of Microbiology, National Taiwan University, Taipei, Taiwan; 2 Institute of Biomedical Sciences, Academia Sinica, Taipei, Taiwan; 3 Department of Computer Science, Iowa State University, Ames, Iowa, United States of America; 4 Department of Clinical Laboratory Sciences and Medical Biotechnology, National Taiwan University, Taipei, Taiwan; 5 CAS Key Laboratory of Pathogenic Microbiology and Immunology, Institute of Microbiology, Chinese Academy of Sciences, Beijing, China; 6 Department of Molecular and Cellular Biology, Center for Cell and Gene Therapy, Stem Cells and Regenerative Medicine Center, Baylor College of Medicine, One Baylor Plaza, BCM505, Alkek Building, N1010.07, Houston, TX 77030, USA

## Abstract

Selection of escape mutants with mutations within the target sequence could abolish the antiviral RNA interference activity. Here, we investigated the impact of a pre-existing shRNA-resistant HBV variant on the efficacy of shRNA therapy. We previously identified a highly potent shRNA, S1, which, when delivered by an adeno-associated viral vector, effectively inhibits HBV replication in HBV transgenic mice. We applied the “PICKY” software to systemically screen the HBV genome, then used hydrodynamic transfection and HBV transgenic mice to identify additional six highly potent shRNAs. Human liver chimeric mice were infected with a mixture of wild-type and T472C HBV, a S1-resistant HBV variant, and then treated with a single or combined shRNAs. The presence of T472C mutant compromised the therapeutic efficacy of S1 and resulted in replacement of serum wild-type HBV by T472C HBV. In contrast, combinatorial therapy using S1 and P28, one of six potent shRNAs, markedly reduced titers for both wild-type and T472C HBV. Interestingly, treatment with P28 alone led to the emergence of escape mutants with mutations in the P28 target region. Our results demonstrate that combinatorial RNAi therapy can minimize the escape of resistant viral mutants in chronic HBV patients.

Patients chronically infected with hepatitis B virus (HBV) typically produce high viral DNA titers^1^,^2^ and viral protein levels^3^,^4^, both of which are associated with a high risk of developing several severe liver complications, including cirrhosis and hepatocellular carcinoma. Nucleoside and nucleotide analogs (NA) have potent antiviral effects by inhibiting the enzymatic function of HBV polymerase, which has reverse transcriptase function, leading to a reduction in serum HBV DNA^5^. However, the lack of proof-reading ability during reverse transcription, combined with a high replication rate, leads to the coexistence of a mixture of genetically distinct, but closely related, viral populations in chronic HBV patients, known as viral quasispecies. For some NA drugs with a low resistance barrier, such as lamivudine, adefovir and telbivudine, long-term treatment often leads to selection from the quasispecies pool of pre-existing drug-resistant viral variants with higher fitness^6^. Moreover, some resistant mutations can confer cross-resistance to other NA drugs, which largely negates the benefits of therapy^7^,^8^,^9^.

RNA interference (RNAi) regulates gene expression by directing mRNA degradation and translational inhibition^10^. RNAi-based therapy is considered as a potentially powerful approach for treating chronic HBV infection that has certain advantages over NA drugs^11^,^12^,^13^. NA drug treatment, although effective in reducing serum HBV DNA levels, has only a limited effect on the high viral antigenemia that is reported to correlate with the progression of liver diseases^3^,^4^ and HBV-specific immune suppression^14^,^15^ in chronic HBV patients. In our previous study, we identified a highly potent anti-HBV small hairpin RNA (shRNA), designated as S1, and demonstrated that a single treatment of HBV transgenic mice with S1 shRNA delivered by adeno-associated viral vector serotype 8 (AAV8) not only reduced the serum HBV titer by up to 1,000- to 10,000-fold, but, more importantly, also substantially decreased serum and hepatic levels of the HBV proteins^12^. The inhibition effect of a single AAV8/S1 treatment lasted for up to 4 months without apparent toxicities^16^. The viral inhibitory effect can be further prolonged by subsequent injection of a different AAV serotype (AAV7 or AAV9) encoding the same S1 shRNA, resulting in reduced liver tumor incidence in aged HBV transgenic mice^17^,^18^.

One major problem for antiviral RNAi therapy is the selection of escape mutants with a point mutation or deletion within the target sequence that could abolish the RNAi activity. This is a particular concern for RNA viruses whose replication polymerases lack proof-reading activity and are prone to mutant generation. In cell culture studies, the therapeutic use of a single shRNA for human immunodeficiency virus^19^, poliovirus^20^ and hepatitis C virus^21^ resulted in rapid emergence of RNAi-resistant viral variants. Selection of RNAi-resistant variants could also be a potential problem in the treatment of chronic HBV patients due to the high error rate of HBV polymerase and the presence of many genetically distinct viral populations in these patients. Indeed, a spontaneously occurring HBV variant which is resistant to the potent S1 shRNA had been identified in a chronic HBV patient^22^. In attempts to overcome this problem, several studies have documented successes using a combinatorial multi-target approach to RNAi therapy^23^,^24^,^25^. For example, treatment with a formulation containing a number of siRNAs, each targeting a unique and highly conserved sequence in the HIV genome, can greatly augment the antiviral effects and prevent the emergence of RNAi-resistant mutants^23^. S1 was the only highly potent shRNA identified in our previous studies using ICR/HBV transgenic mice, a more clinically relevant HBV animal model that produce and maintain more than 10^8^ HBV virions/ml of serum^12^. In this report, our first aim was to identify more HBV-targeting shRNAs with high potency *in vivo* that would allow us to perform combinatorial RNAi therapy. We then used human liver chimeric mice as a HBV infection model to evaluate the therapeutic efficacy, and monitor the dynamics of HBV variants during treatment with a single shRNA or a combination of different shRNAs.

## Results

### A mutant HBV resists RNAi therapy with S1

**Figure 1:**
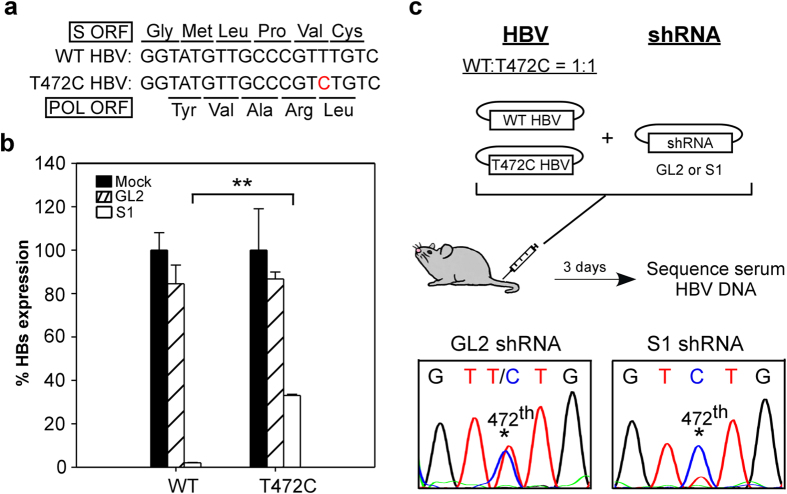
T472C HBV is partially resistant to S1 shRNA. (**a**) The nucleotide sequence and corresponding amino acid sequence within the S1 target site of wild type (WT) and T472C HBV genome. The triplet codons for surface and polymerse open reading frame (ORF) are illustrated by lines located above and below the sequence, respectively. The mutated nucleotide in T472C HBV is shown in red, which does not change the amino acid sequence in either surface or polymerase genes. (**b**) Reduction in HBsAg levels in cell culture medium by shRNAs. Huh-7 cells were co-transfected with shRNA- and wild-type HBV (WT, pHBV1.3) or T472C mutant HBV-encoding plasmids. Mock transfection served as negative controls. Three days later, HBsAg levels in the supernatant were measured by ELISA and are presented as a percentage of those with mock transfection (each value represents the mean ± SD of three independent transfection experiments). **P < 0.01. (**c**) Schemati**c** diagram of hydrodynamic co-injection used to analyze the S1-resistance of T472C HBV. C57BL/6 mice (n = 7 per group) were hydrodynamically co-injected with equal amounts of WT and T472C HBV plasmids and a plasmid encoding either S1 or GL2 shRNA, then, 3 days later, serum HBV DNA was extracted for PCR amplification and the ratio of the two HBV strains estimated by sequencing the PCR product corresponding to the S1 target region. * indicates the position of nt 472. (Picture credit: Y.M.S.)

The T472C HBV variant that carried a T to C silence mutation within the S1 target site was originally identified in a chronic HBV patient who had not received RNAi treatment (Fig. 1a)^22^. The T to C mutation does not change the amino acid sequences of either surface or polymerase genes. We generated T472C HBV by site-directed mutagenesis of the pHBV1.3 plasmid, which carries a 1.3-fold wild-type HBV genome, then co-transfected Huh-7 cells with the wild-type or T472C HBV plasmid and a pSuper plasmid encoding shRNA S1 or GL2 (an irrelevant shRNA). As shown in Fig. 1b, treatment with S1 resulted in a marked reduction in HBsAg levels in the medium from cells transected with wild-type HBV, but only a moderate reduction with cells transfected with T472C HBV. The irrelevant GL2 shRNA had no effect on HBsAg levels with either HBV strain.

We then assessed whether T472C HBV could escape from RNAi treatment *in vivo*. We hydrodynamically co-injected C57BL/6 mice with a 1:1 mixture of wild-type and T472C HBV plasmids and either the S1- or GL2 shRNA-expressing plasmid, then, after 3 days, extracted serum HBV DNA and determined the relative amounts of wild-type and T472C HBV by sequencing. As shown in Fig. 1c, in the S1 shRNA-treated mice, T472C HBV became the predominant virus in the population, while, in control GL2 shRNA-treated mice, the ratio of wild-type to T472C HBV remained unchanged. These results demonstrate that the *in vivo* HBV suppressive effect of shRNAs is highly sequence-dependent and that the presence of a shRNA-resistant HBV variant, such as HBV T472C, can significantly reduce the efficacy of RNAi therapy, resulting in drug-resistant viral escape.

### Screening of highly potent HBV-targeting shRNAs predicted using the PICKY software

In order to apply combination therapy using multiple shRNAs, we set out to identify additional shRNAs with a silencing efficacy comparable to, or better than, that of S1. We had previously screened more than 10 different shRNAs designed by the widely used Reynolds criteria^26^ or reported in the literature to be effective in silencing HBV gene expression *in vivo*^27^ and found S1 is the only shRNA that can achieve sustained HBV suppression in ICR/HBV transgenic mice (12,16 ; C.C. Chen, unpublished data). To increase the chance of identifying shRNAs with a high potency *in vivo*, we used the PICKY software to analyze the HBV genome and select potential powerful HBV-targeting shRNA candidates that not only fitted the shRNA criteria, but also showed low non-specific binding to host RNAs, thus increasing the chance of targeting the HBV transcripts^28^,^29^.

**Figure 2:**
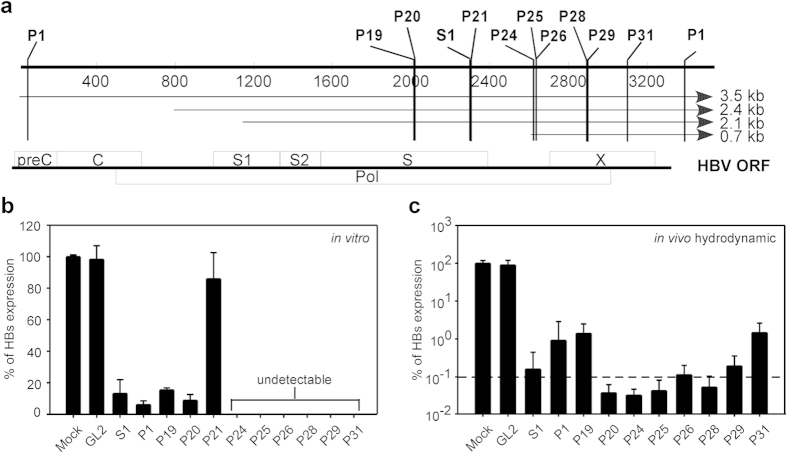
Screening of PICKY-predicted shRNAs by co-transfection of HBV- and shRNA-encoding plasmids. (**a**) Schematic representation of the HBV genome (bold line), the four transcripts (thin lines), and the open reading frames (ORF, boxes). The approximate locations of the target sequences predicted by the PICKY software are indicated. (**b**) Huh-7 cells were transfected with wild-type HBV plasmid alone or together with a plasmid encoding the indicated shRNA (each value represents the mean ± SD of three independent transfection experiments) or (**c**) C57BL/6 mice (n = 5–7) were hydrodynamically injected with wild-type HBV plasmid alone or together with a plasmid encoding the indicated shRNA, then, 3 days later, HBsAg levels in the culture supernatant (**b**) or serum (**c**) were measured. The data are presented as a percentage of the level produced by cells transfected with HBV plasmid alone (**b**) or mice injected with HBV plasmid alone (mock) (**c**) (mean ± SD).

PICKY predicted 31 HBV-targeting shRNAs with high scores. From these, we chose 10 that each would target at least 3 of the 4 HBV transcripts and the sequences of which were conserved in at least 3 of the 8 HBV genotypes. The target regions of these PICKY-selected shRNAs in the HBV genome are shown in Fig. 2a. We first cloned the PICKY-predicted shRNA sequences into the pSuper plasmid, then co-transfected Huh-7 cells with equal amounts of pHBV1.3 and a pSuper plasmid encoding one of the selected shRNAs and measured HBsAg levels in the culture supernatant 3 days later. As shown in Fig. 2b, 9 of the 10 PICKY-selected shRNAs (exception P21) markedly reduced HBsAg levels. Three (P1, P19, and P20) reduced HBsAg levels by ~90%, while the other six (P24, P25, P26, P28, P29, and P31) were even more effective and reduced HBsAg levels to background. To examine the *in vivo* antiviral effect of these shRNAs, we hydrodynamically co-injected each pSuper plasmid and pHBV1.3 at a 3:1 ratio into C57BL/6 mice and measured serum HBsAg levels at day 3 post-injection. As shown in Figs 2c and 6 of these shRNAs (P20, P24, P25, P26, P28, and P29) were as effective as S1 in reducing HBsAg levels, while P1, P19, and P31 were less potent.

**Figure 3:**
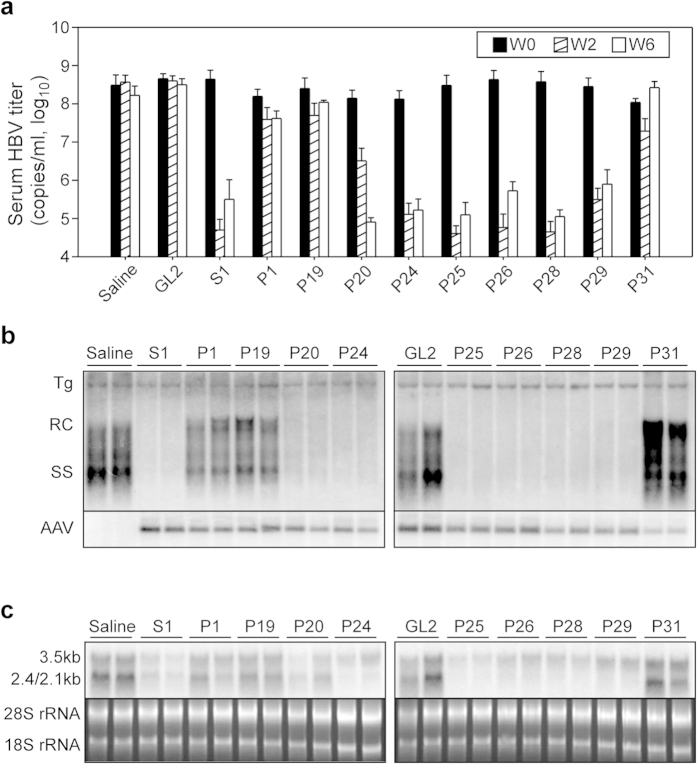
Screening of PICKY-predicted shRNAs in HBV transgenic mice. ICR/HBV mice (n = 3–5) were injected i.v. with 1 × 10^12^ vg per mouse of AAV8 vector encoding the indicated shRNA or saline. (**a**) Serum s**a**mples were collected before treatment and at 2 or 6 weeks after treatment and the HBV DNA titer determined (mean ± SD). (**b,c**) The mice were sacrificed at week 6 and liver tissues collected for (**b**) Southern or (**c**) Northern blot analysis. (**b**) Total liver DNA was analyzed for HBV replicative intermediates (upper panel) and AAV vector genomes (lower panel). Bands corresponding to the integrated transgene (Tg) and the relaxed circular (RC) or single-stranded (SS) linear HBV DNA replicative form are indicated. The integrated transgene was used to normalize the amount of DNA loaded on the gel. (**c**) Total liver RNA was analyzed for the 3.5 kb and 2.4/2.1 kb HBV transcripts (upper panel); ethidium bromide-stained 18 S and 28 S ribosomal RNA (rRNA) served as the loading controls (lower panel).

We then packaged each of the PICKY-selected shRNA into the AAV8 vector and injected groups of HBV transgenic mice (n = 5) i.v. with 1 × 10^12^  vg per mouse of AAV8 expressing each of the PICKY-selected HBV-targeting shRNAs, using AAV8 vectors expressing, respectively, S1 or GL2 shRNA as positive or negative controls, and measured the serum HBV titer in each group before, and two and six weeks after, AAV administration. As shown in Fig. 3a, AAV8/P24, P25, P26, P28, or P29 profoundly reduced the serum HBV DNA titer by more than 900-fold (range 903- to 8,463-fold) at week 2 and by more than 350-fold (range 358- to 3,353-fold) at week 6, the level of HBV suppression being comparable to that seen with AAV8/S1 (8,795-fold at week 2 and 1,134-fold at week 6). AAV8/P20 showed a slow antiviral effect, reducing the serum HBV titer by only 43-fold at week 2, but by 1,737-fold at week 6. In contrast, AAV8/P1, P19, or P31 had little HBV suppressive effect (less than 5-fold at week 2 or 6). We chose three promising shRNAs, AAV8/P25, P26, and P28, to conduct a long-term kinetic study and showed that AAV8/shRNA treatment resulted in a marked decrease in both HBV DNA titers and HBsAg levels and, importantly, the suppressive effects sustained for at least 16 weeks post-injection (Supplementary Fig. S1).

We then sacrificed these AAV8/shRNA-treated mice at week 6 and measured levels of liver HBV DNA and RNA, respectively, by Southern or Northern blots. As shown in the upper panel of Fig. 3b, use of AAV8/P20, P24, P25, P26, P28, or P29 resulted in undetectable levels of relaxed circular (RC) and single-stranded linear (SS) HBV DNA, a result comparable to that seen in the AAV8/S1 group, while AAV8/P1, P19, or P31 had only a minor effect on intrahepatic HBV DNA levels. As shown in the lower panel of Fig. 3b, Southern blot analysis of intrahepatic AAV DNA levels in the different groups showed that the different HBV suppressive effects of the PICKY-selected shRNAs was not due to different transduction efficiencies of the AAV vectors. In the Northern blot analysis, shown in Fig. 3c, we normalized HBV mRNA levels to those of the internal control 28S/18S rRNA, then compared the suppressive effect of each shRNA to that of the saline group and found that AAV8/P20, P24, P25, P26, P28, or P29 was as effective as AAV8/S1 in reducing HBV transcripts, while AAV8/P1, P19 and P31 were much less potent. We chose three potent shRNAs (P25, P26 and P28) and three weak shRNAs (P1, P19, P31) to measure expression levels of the antisense strand of each processed shRNA using specific isotope-labeled oligonucleotide probes. Consistent with their anti-HBV activity, mice injected with AAV8/P25, P26 or P28 expressed much more mature guide RNA than mice injected with AAV8/P1, P19 or P31 (Supplementary Fig. S2).

In our previous reports, we showed that AAV8/S1 treatment was able to achieve sustained HBV DNA and protein suppression without showing liver damage or apparent toxicity^16^. We carried out a set of experiments to examine the toxicity profiles of the three potent PICKY-selected shRNAs, P25, P26, and P28. We first investigated whether exogeneous expression of these shRNAs by AAV8 vectors would saturate the natural microRNA pathway and cause liver toxicity^27^. Liver tissues from HBV transgenic mice injected with shRNA-expressing AAV8 vectors (P25, P26, P28, S1 or GL2) or saline as described above were analyzed by quantitative reverse transcription-PCR (RT-qPCR) to measure the microRNA-122 level, which is known to be specifically expressed in the liver. As shown in Supplementary Fig. S3a, microRNA-122 expression in the liver tissues of mice injected with any of the AAV8/shRNA vectors was indistinguishable from that in the saline control animals. We next examined whether IFN-α/β, IFN-γ and TNF-α, which were known to inhibit HBV DNA replication in transgenic mice^30^, were induced by the three PICKY selected shRNAs using RT-qPCR. We measured 2′–5′-oligoadenylate synthetase 1 (OAS1) because it is a major target gene induced by IFN-α/β. As shown in Supplementary Fig. S3b, only negligible amounts of OAS1 and TNF-α and no IFN-γ mRNAs were present in the liver samples of mice treated with saline or the AAV8 vectors. In contrast, HBV transgenic mice injected with a recombinant adenovirus carrying the mouse interleukin 12 gene produced high levels of OAS1, IFN-γ and TNF-α. Consistent with the results of microRNA and cytokine analysis, in all AAV8/shRNA groups, serum alanine aminotransferase (ALT) levels, an indicator of liver injury, remained within the normal range (Supplementary Fig. S3c) and no apparent pathological changes were observed in the liver tissues by histological analysis (Supplementary Fig. S3d). These data demonstrate that these PICKY-selected shRNAs have good safety profiles for *in vivo* application.

### Prevention of viral escape in human liver chimeric mice by combinatorial shRNA treatment

**Figure 4:**
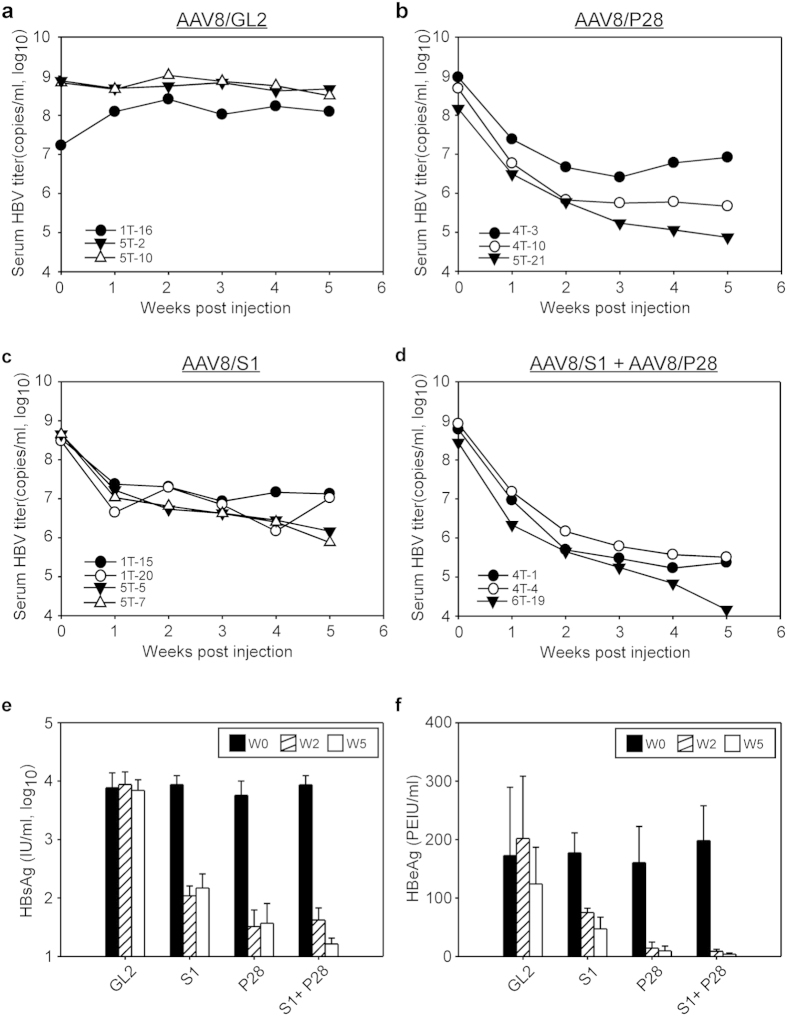
Evaluation of the therapeutic efficacy of AAV-mediated shRNAs in HBV-infected hu-FRG mice. (**a–d**) HBV-infected hu-FRG mice (n = 3–4) were injected i.v. with 1 × 10^12^ vg per mouse of AAV8 vector encoding (**a**) GL2, (**b**) P28, (**c**) S1, or (**d**) S1 + P28 shRNA, then serum samples were collected at different times and the HBV DNA titer determined by qPCR. The identification numbers of the animals in each group are shown in the lower left corner of each panel. (**e**,**f**) Levels of HBsAg (**e**) and HBeAg (**f**) in the serum samples were determined before treatment and at 2 or 5 weeks after treatment (mean ± SD).

Human liver chimeric mice can be stably infected by HBV^31^,^32^,^33^ and can, theoretically, support the replication of heterogeneous HBV quasispecies, providing a convenient animal model for preclinical drug evaluation. We used human hepatocyte-transplanted *Fah*^*−/−*^*/Rag2*^*−/−*^*/Il2rg*^*−/−*^ (hu-FRG) mice to investigate the efficacy of anti-HBV shRNAs in the context of human hepatocytes and the impact of a pre-existing resistant HBV variant on shRNA therapy. We first demonstrated that wild-type and T472C HBV displayed similar kinetics of serum viremia in hu-FRG mice (Supplementary Fig. S4), indicating that T472C HBV did not have a defect in viral replication. Then 13 hu-FRG mice with circulating human albumin levels of at least 1 mg/ml were infected with a 9:1 mixture of wild-type and T472C HBV to mimic the heterogeneous population of HBV in the chronic HBV patient in whom T472C HBV was identified^22^. At 6–8 weeks post-infection, these mice displayed a median viremia level of 4.5 × 10^8^ HBV DNA copies/ml (range 1.7 × 10^7^ to 9.4 × 10^8^ copies/ml) and were randomly assigned to different groups (n = 3–4) and injected i.v. with AAV8 vectors expressing GL2, S1, or P28 (1 × 10^12^ vg per mouse) or with vectors expressing S1 and P28 (each 5 × 10^11^ vg per mouse), then serum samples were collected weekly to measure HBV titer. As shown in Fig. 4a, control AAV8/GL2 treatment of 3 hu-FRG mice had little effect on the serum HBV DNA titer, all having >1 × 10^8^ copies/ml of serum HBV DNA at the end of the 5-week observation period. Figure 4b shows that AAV8/P28 treatment of 3 mice resulted in an average 56-fold reduction at 1 week after treatment compared to the pretreatment value and that two mice showed a continuous decrease in serum HBV DNA titer, with reductions of 1,041-fold (mouse 4T-10) and 1,974-fold (mouse 5T-21) at week 5; interestingly, mouse 4T-3 showed a different pattern of HBV inhibition, with a peak reduction (366-fold) in HBV titer at week 3, followed by a rebound of viremia, with a 113-fold reduction in HBV DNA titer at week 5. Figure 4c shows that, in the 4 mice treated with AAV8/S1, an average 38-fold reduction in HBV DNA titer was seen 1 week after treatment, followed by an increase in inhibitory effect in some mice, but not others; at week 5, two mice (1T-15 and 1T-20) displayed a weak reduction (about 30-fold) in serum HBV DNA titer, while the other two showed a modest reduction (307-fold in mouse 5T-5 and 592-fold in mouse 5T-7). Significantly, as shown in Fig. 4d, combinatorial treatment with AAV8/S1 and AAV8/P28 had the best therapeutic effect against HBV in 3 mice even in the presence of a pre-existing variant, resulting, at the end of the 5-week treatment, in a 2,572- and 2,628-fold reduction, respectively, in mouse 4T-1 and 4T-4 and almost undetectable levels in mouse 6T-19. Decrease of the HBV DNA titer in these human liver chimeric mice was not due to loss of human hepatocytes because human albumin levels in these different groups of HBV-infected hu-FRG mice did not change significantly over the experimental period (Supplementary Fig. S5).

We confirmed the therapeutic effect of AAV-delivered shRNAs in hu-FRG mice by measuring HBV protein levels (HBsAg and HBeAg) in the sera of the same treated mice. Figure 4e,f show, respectively, average levels of HBsAg or HBeAg before treatment and at 2 or 5 weeks after treatment. AAV8/GL2 treatment had no effect on serum HBsAg and HBeAg levels over the treatment period. AAV8/S1 treatment led to a modest reduction in HBsAg levels (80-fold reduction at week 2 and 59-fold reduction at week 5) and in HBeAg levels (2-fold reduction at week 2 and 4-fold reduction at week 5), while AAV8/P28 treatment had a greater inhibitory effect on HBsAg levels (176-fold reduction at week 2 and 155-fold reduction at week 5) and HBeAg levels (11-fold reduction at week 2 and 17-fold reduction at week 5). However, the greatest inhibitory effect was seen in the combination treatment group (206-fold reduction in HBsAg levels at week 2 and 531-fold reduction at week 5; 23-fold reduction in HBeAg levels at week 2 and 53-fold reduction at week 5).

**Figure 5:**
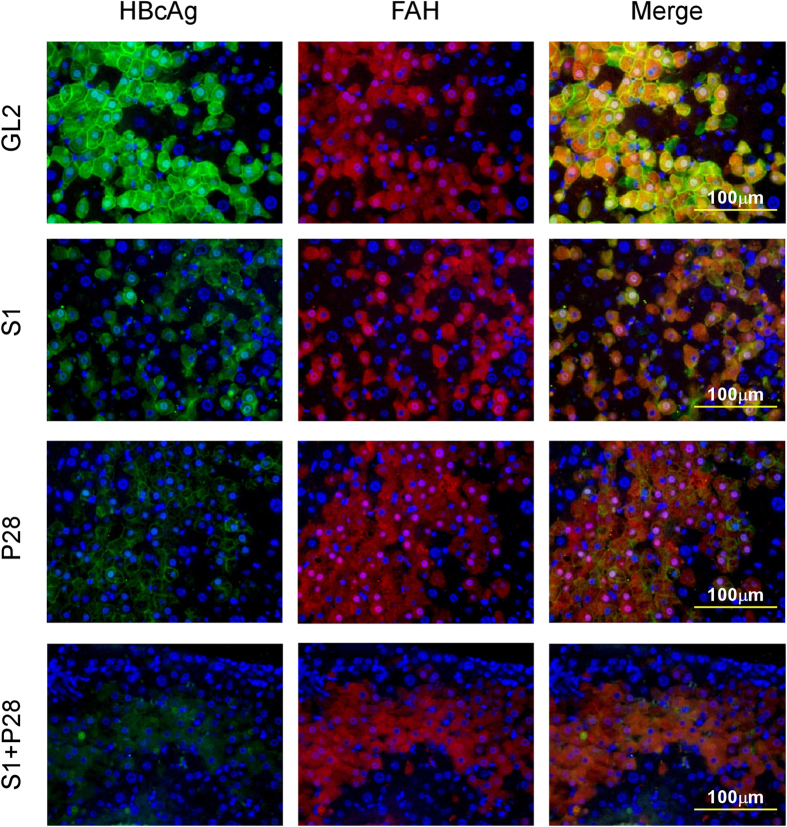
Effect of shRNA treatment on intrahepatic HBcAg in HBV-infected hu-FRG mice. HBV-infected hu-FRG mice treated with different AAV/shRNAs as described in Fig. 4 were sacrificed at week 5 post AAV injection and liver samples obtained for immunofluorescent staining. Human hepatocytes were visualized by red staining of human fumarylacetoacetate hydrolase (FAH) (middle panels), HBcAg staining was shown in green (left panels), and DAPI (blue) was used as nuclear counterstaining. The merged pictures show HBcAg expression in human hepatocytes (yellow; right panels). Original magnification, × 400; bar, 100 μm.

These AAV8/shRNA-treated mice were sacrificed at week 5 after AAV injection and liver tissues collected for HBcAg analysis by immunostaining and intracellular HBV DNA and RNA analysis by Southern and Northern blotting, respectively. As shown in Fig. 5, in the control AAV8/GL2 group almost all human hepatocytes were infected by HBV and showed strong HBcAg staining. Among the three HBV shRNA-treated groups, combination treatment with AAV8/S1 plus AAV8/P28 had the greatest effect on HBcAg levels, while treatment with AAV8/S1 alone had the least effect. Southern blots (Supplementary Fig. S6a) showed that treatment with S1, P28, or S1+P28 shRNAs reduced levels of RC and SS HBV DNA to almost undetectable, and Northern blots (Supplementary Fig. S6b) showed that 3.5 kb and 2.4/2.1 kb HBV transcript levels were reduced in all three HBV shRNA-treated groups.

### Resistant mutation occurred in serum HBV but not in intrahepatic cccDNA in mono-shRNA treated mice

**Figure 6:**
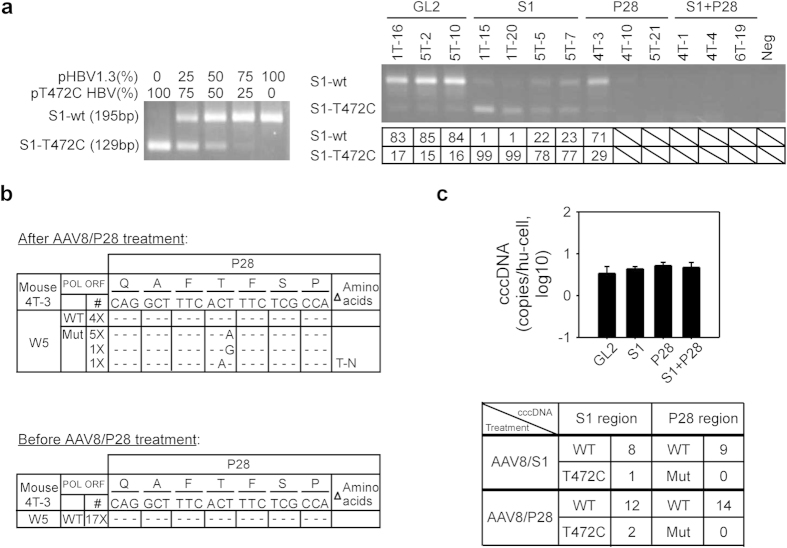
HBV variant analysis in AAV8/shRNA-treated hu-FRG mice. (**a**) Left panel: Setting up of the PCR-RFLP assay for rapid discrimination of wild-type and T472C HBV. The wild-type and T472C HBV plasmids were mixed at the indicated ratios and digested with BccI to generate different ratios of the S1-wt (195 bp) and S1-T472C (129 bp) fragments for use as a standard. Right panel: Serum samples collected at 5 weeks after AAV8/shRNA treatment from the huFRG mice described in Fig. 4 were analyzed by PCR-RFLP assay and the DNA fragments separated on a 4% agarose gel. The faster migrating band indicates the T472C HBV sequence (S1-T472C) and the slower migrating band indicates the wild-type HBV sequence S1-wt. (**b**) Sequence analysis of the P28 target regions in the serum HBV genome from mouse 4T-3 at 5 weeks after (top panel) and before (bottom panel) AAV8/P28 treatment. (**c**) Analysis of intrahepatic cccDNA. Liver samples collected at 5 weeks post AAV8/shRNA treatment from the huFRG mice described in Fig. 4 were used in a real-time qPCR assay, using selective cccDNA primers and FRET hybridization probe, to quantify the intrahepatic cccDNA levels. The amount of human mitochondria DNA was quantified using specific primers and used to normalize cccDNA copies (mean ± SD) per human hepatocyte (expressed as human genome equivalents) determined in chimeric livers (top panel). Sequence analysis of the shRNA target regions in the intrahepatic cccDNA extracted from AAV8/S1 or AAV8/P28 treated mice. The number of clones with wild-type and mutations in the S1 and P28 regions are shown (bottom panel).

To investigate whether a pre-existing T472C HBV variant could escape the effect of S1 shRNA treatment and become the dominant strain, we used PCR and restriction fragment length polymorphism (PCR-RFLP) analysis to rapidly distinguish between wild-type and T472C HBV in serum samples and quantify the ratio. The DNA fragment covering the S1-targeted region was amplified by PCR and introduced into a BccI site generated in the PCR fragment of T472C HBV, but not wild-type HBV; BccI digestion of the T472C PCR fragment would therefore generate two small fragments of 129 bp (S1-T472C) and 66 bp (too small to be visualized on the gel), whereas the 195 bp fragment (S1-wt) from wild-type HBV would not be cut. We first prepared mixtures of S1-wt and S1-T472C at different ratios and used them as standards to estimate the ratio of the two HBV strains in test samples (Fig. 6a, left panel), then analyzed the serum DNA samples from the HBV-infected hu-FRG mice described in Fig. 4 at 5 weeks post-AAV injection. As shown in the right panel of Fig. 6a, in mice treated with the control AAV8/GL2, the HBV consisted of 84% S1-wt DNA and 16% S1-T472C, close to the input ratio of 9:1. AAV8/S1 treatment markedly decreased the proportion of S1-wt from 90% to 1–23% and thus greatly increased that of S1-T472C from 10% to 77–99%, showing that a shRNA-resistant HBV variant was able to dominate the viral population under the selection pressure of shRNA treatment. Since both wild-type and T472C HBV were sensitive to P28 shRNA treatment, treatment with AAV8/P28 alone or AAV8/P28 plus AAV8/S1 reduced the DNA levels for both viral strains to an undetectable level in all mice except mouse 4T-3, which still exhibited a significant amount of HBV DNA, with 71% S1-wt DNA and 29% S1-T472C HBV DNA.

To determine whether the viral relapse in mouse 4T-3 was due to the emergence of mutations within the P28 target region, we amplified HBV DNA in the serum samples collected at 5 weeks post-treatment by PCR, cloned it into a plasmid, and analyzed it by sequencing, with a particular focus on the P28 and S1 target regions. In the P28 target region, 4 out of 11 clones showed the wild-type sequence (36%) and 7 showed one nucleotide mutation (64%), with 6 having silent mutations of T-to-A or T-to-G at position 12 and one carrying a mutation at position 11, resulting in an amino acid change from Thr to Asn (Fig. 6b, top panel). In the S1 target region, which was not under selection pressure in mouse 4T-3, 10 clones exhibited the wild-type sequence and 1 the T472C sequence, a ratio close to the initial input ratio of wild-type and T472C HBV. To prove that the mutations in the P28 target region were induced during AAV8/P28 treatment, we also analyze the serum samples collected before treatment. In these samples, the ratio of wild-type to T472C HBV was 15:2 in the S1 target region (data not shown), and, as expected, all clones had the wild-type sequence in the P28 target region (Fig. 6b, bottom panel). Furthermore, in the 5 week samples collected from the GL2 shRNA-treated mice, no mutations were seen in the P28 target region and the ratio of wild-type to T472C in the S1 target region was the same as the input ratio (data not shown). Together, these results show that the HBV variants that dominated in the week 5 sample of the AAV8/P28-treated mouse were generated under the selection pressure of P28 shRNA.

Given that covalently closed circular DNA (cccDNA) is the transcriptional template for HBV, we determined whether RNAi treatment has an effect on the intrahepatic cccDNA level and whether the cccDNA in the S1 and P28 mono-therapy mice, which saw dominant shRNA-resistant variants in the serum, contains mutations in the shRNA target region. We measured the cccDNA levels among the different groups using a validated cccDNA-specific quantitative polymerase chain reaction (qPCR) and normalized to human genomic DNA. As shown in the top panel of Fig. 6c, we did not see significant difference of the amounts of cccDNA in mice treated with AAV8 vectors expressing S1, P28 or S1+P28 as compared to that in the AAV8/GL2 control. The intrahepatic levels of cccDNA in all groups were between 3 and 5 copies per cell. We then PCR amplified the DNA fragments covering the S1 and P28 region from the cccDNA of mice treated with S1 or P28 and cloned them into plasmids. Sequencing analysis of the S1 region revealed that treatment with S1 or P28 had little effect on the ratio of wild-type to T472C in the cccDNA, 8:1 in the S1 treatment group and 12:2 in the P28 treatment group, which were close to the initial input ratio of 9:1 (Fig. 6c, bottom panel). Similarly, S1 and P28 monotherapy did not induce detectable mutations in the P28 target region in the cccDNA (Fig. 6c, bottom panel), even in mouse 4T-3 which was treated with P28 and showed selection of a dominant viral variant in the serum with a T-to-A or T-to-G point mutation at residue 1097.

## Discussion

A major challenge in RNAi-based therapeutics is the development of escape mutant viruses^19^,^20^,^21^,^22^. In this study, we demonstrated that a HBV variant, T472C HBV, identified in the pre-existing quasispecies pool of a chronic HBV patient, was able to escape from S1 treatment and become the dominant population *in vivo* (Figs 1 and 4). Although T472C HBV was not commonly found in chronic HBV patients (data not shown), we decided to use it as a model system to test the ability of combinatorial RNAi therapy to prevent viral escape and therefore set out to identify other potent shRNAs in addition to S1. In this study, we used the “PICKY” software to predict potential siRNA candidates from the entire HBV genome. In the more stringent ICR/HBV transgenic mouse model, we used AAV8 vectors to express these candidate shRNAs, and found that P20, P24, P25, P26, P28, and P29 were able to achieve up to an 8,000-fold reduction in HBV DNA titer and 100-fold reduction in HBsAg level (Fig. 3a and Supplementary Fig. S1), results comparable to that using S1. Analysis of intracellular HBV replication intermediates revealed that the use of any one of these 6 PICKY-selected shRNAs resulted in almost background levels of HBV DNA and greatly reduced HBV RNA levels at week 6 post-injection (Fig. 3b,c). Although we did not test the remaining 21 PICKY-selected shRNA candidates targeting HBV, our results suggest that many of them might also be useful for combinatorial RNAi treatment. The RNAi therapeutic effect might be further improved by using a recently developed chimeric AAV serotype with highly efficient transduction efficiency of human hepatocytes in a human liver chimeric mouse model^34^.

A particular concern with the RNAi-based approach is the off-target effect that may cause *in vivo* toxicity. We show that administration of AAV8/shRNA at a dose of 1 × 10^12^ vg per mouse was well tolerated without elevation of liver enzymes (Supplementary Fig. S3c) or evidence of liver toxicity (Supplementary Fig. S3d). Injection of AAV8/shRNA vectors did not induce detectable IFN-α/β, IFN-γ and TNF-α (Supplementary Fig. S3b) or interfere with microRNA biogenesis (Supplementary Fig. S3a), excluding the possibility that HBV inhibition in these shRNA-treated animals was through the anti-viral effects of these inflammatory cytokines^30^ or off-target effects associated with endogeneous microRNA dysregulation. To further prove that the silencing effect of shRNAs is target sequence-dependent, we used site-directed mutagenesis to generate a HBV variant with a T-to-A point mutation at residue 1097 (T1097A) in the P28 targeting region, which is the dominant HBV strain emerging after AAV8/P28 treatment (Fig. 6b, top panel). In a hydrodynamic co-injection experiment, we found that a single point mutation in the T1097A HBV variant resulted in complete resistance to P28 shRNA (Supplementary Fig. S7). Together, these results support that the silencing effect of these PICKY-selected shRNAs is due to a direct effect on HBV transcripts but not through an off-target effect on host transcripts.

Treatment of NA drugs with low resistance barrier often leads to selection from the quasispecies pool of pre-existing drug-resistant viral variants with higher fitness, resulting in treatment failure^35^,^36^. We used hu-FRG mice to carry out a proof-of-principle preclinical study to evaluate the antiviral effect of AAV-mediated RNAi therapy in the context of human hepatocytes and the impact of pre-existing HBV quasispecies on the effectiveness of RNAi therapy. To mimic the heterogeneous HBV population in clinical patients, we inoculated hu-FRG mice with a 9:1 mixture of wild-type HBV and T472C HBV, a HBV mutant resistant to S1 shRNA, then, when the viral load reached a steady high level, treated them with AAV8/shRNA. Our results showed that AAV8/S1 treatment resulted in a reduction in serum HBV DNA titer of between 40- and 592-fold as compared to the pretreatment value (Fig. 4c). Importantly, we found that the pre-existing shRNA-resistant T472C mutant was selected and became the dominant strain in the serum of this treatment group. As shown by PCR-RFLP assay, in 2 of the 4 mice treated with AAV8/S1, wild-type HBV was completely replaced by T472C HBV within the 5-week observation period, while the other 2 mice exhibited an increase in the proportion of T472C HBV from 10% to about 80% (Fig. 6a). The replacement of wild-type HBV by T472C HBV was not due to the replication advantage of T472C HBV, because mice mono-infected with one of the two HBV strains showed similar viral kinetics (Supplementary Fig. S4). Interestingly, while T472C HBV became the dominant viral strain in the serum 5 weeks after S1 treatment, we did not see a significant change of the ratio of wild-type to T472C mutation in the intrahepatic cccDNA (Fig. 6c, bottom panel). This is probably due to the stability and long half-life of HBV cccDNA^37^,^38^,^39^. These results strongly suggest that T472C HBV had a survival advantage under the selection pressure of S1 shRNA treatment and quickly became the dominant viral strain in the serum but may require a longer time to completely replace wild-type HBV in the intrahepatic level.

In several *in vitro* cell culture studies, it has been reported that shRNA escape mutants were rapidly selected under the selection pressure of RNAi treatment of several RNA viruses^20^,^21^,^23^. In this study, we investigated whether this phenomenon also occurred in HBV, a DNA virus with a high mutation rate, using a more clinically relevant *in vivo* animal model. We showed that of the 3 hu-FRG mice treated with P28 shRNA, which is fully complementary to the transcripts of both wild-type and T472C HBV, mouse 4T-3 displayed a peak reduction of only 366-fold at week 3, then experienced a rebound of viremia (Fig. 4b). To determine whether P28-specific mutations were selected under the pressure of P28 shRNA treatment, we PCR-amplified and sequenced the P28 region of the HBV genome isolated from mouse 4T-3, using the non-targeted S1 region as a control, and found that 7 out of 11 (64%) clones contained mutations in the P28 target region, while no mutations other than the input T472C HBV mutation were identified in the non-targeted S1 region (data not shown). These results suggest that treatment with a single shRNA is not able to achieve long-term suppression of HBV, similar to what have been reported for RNA viruses with high error-prone replication^20^,^21^,^23^, because of the selection of pre-existing shRNA-resistant variants from the quasispecies population or the generation of new mutant strains during the ongoing infection and selection under shRNA pressure.

To prevent selection of RNAi-resistant HBV mutants, we used combination therapy with S1 and P28 shRNAs to treat hu-FRG mice co-infected with wild-type HBV and T472C HBV and achieved a better inhibition than with either of the single therapies, resulting in a more than 2,500-fold peak reduction in serum HBV DNA titers in two mice and complete elimination in the other (Fig. 4d). Importantly, no viral rebound was observed in these mice during the experimental period (5 weeks). These results strongly suggest that combinatorial therapy with two or more shRNAs that target different locations in the HBV genome can reduce the chance of viral escape and maintain therapeutic efficacy. However, it should be noted that AAV8/shRNA treatment, although achieving a near complete clearance of liver HBV DNA and RNA (Supplementary Fig. S6), had very little effect on the intrahepatic cccDNA levels (Fig. 6c, top panel). Thus, it is likely that the shRNA-mediated silencing effect might gradually decrease with time due to the decrease of AAV genome copy number in the liver^12^. In this regard, sequential use of different AAV serotypes would be required to prolong the shRNA-mediated HBV suppression^17^. An alternative approach is to use a highly potent shRNA, such as HBV-S1, and a highly potent NA drug, such as tenofovir or entecavir, as combination partner to achieve long-term and complete HBV suppression, minimizing the risk of selecting resistant variants. The unique activity of shRNA in inhibiting HBV transcripts and hence decreasing protein production could further help HBeAg and HBsAg seroconverson during long-term follow-up.

In this study, we used two AAV vectors each expressing a different shRNA for the combinatorial therapy. For convenience, one can simultaneously express multiple shRNAs in a single viral vector by combining multiple shRNA-expression cassettes containing the same or different promoters^23^,^40^. Alternatively, one can also apply long hairpin RNAs^25^ or polycistronic microRNAs^41^ to generate multiple siRNAs in a single cell. These novel delivery vectors, in particular the one with the microRNA backbone, may further improve the safety profiles *in vivo* by reducing siRNA transcription but still maintaining their silencing activity. In this regard, the combinatorial use of a multiple shRNA-delivery vector and the highly potent shRNAs identified in this study might provide a potential cure for, or long term management of, chronic HBV infection.

## Materials and Methods

### PICKY screening

The PICKY screening process (http://www.complex.iastate.edu/download/Picky/shRNA_Design/index.html) includes two major steps to select potential shRNA candidates that are potent and gene-specific. In the first step, all shRNAs are generated which satisfy the following common selection criteria including (i) an A base at the 3′-end of the sense strand; (ii) a G base at the 5′-end of the sense strand; (iii) a T base at base position 10 of the sense strand and (iv) no G base at position 13 of the sense strand^26^. In the second step, PICKY determines the thermodynamic properties of these shRNAs based on the difference of the binding affinity of each shRNA to its selected HBV target sequence and to any close non-target genes in the mouse and human transcriptomes. Note that high binding affinity of a shRNA candidate to its intended HBV target sequences does not necessarily prevent it from also having high binding affinity to many non-target genes if its target region is not thermodynamically unique. Increasing the difference in binding affinity between shRNA target and non-target sequences can theoretically reduce non-specific binding to host RNAs which may cause side-effects and increase the chance of targeting directly to the HBV transcripts^28^,^29^.

### Animals and cell lines

Female C57BL/6 mice at 6–8 weeks of age were purchased from the National Laboratory Animal Breeding and Research Center, Taipei, Taiwan. The NOD.Cg-*Prkdc*^*scid*^ *Il2rg*^*tm1Wjl*^/SzJ (*Nod-scid/Il2rg*^*−/−*^, commonly known as NSG) mice were originally obtained from the Jackson Laboratory (Bar Harbor, Maine, USA). The generation and characterization of the HBV transgenic mice on the ICR background have been reported previously^12^,^18^. This HBV transgenic mouse line produces high levels of serum HBV DNA and proteins, a condition closely mimicking that in progressive chronic HBV-infected patients. We used transgenic mice matched by age (8–12 weeks), sex (male), and serum HBV titer- (1 × 10^8^ genome copies/ml) in all the shRNA treatment studies. *Fah*^*−/−*^*/Rag2*^*−/−*^*/Il2rg*^*−/−*^ (FRG) mice were generated by crossing *Fah*^*−/−*^ mice to *Rag2*^*−/−*^*/Il2rg*^*−/−*^ mice which are completely lack of adaptive immune cells (T lymphocytes and B lymphocytes) but still have some innate immune cells such as macrophages/Kupffer cells in the liver^42^,^43^. All animal studies were conducted in specific pathogen-free conditions and in accordance with *the Guide for the Care and Use of Laboratory Animals*. The protocols used in this study were approved by Academia Sinica Institutional Animal Care and Usage Committee (ASIACUC permit number 12-12-466). Huh-7 cells (kindly provided by Dr. Chiaho Shih, Academic Sinica), a human hepatoma cell line, were maintained in RPMI-1640 medium (Sigma-Aldrich, St Louis, MO) supplemented with heat-inactivated 10% fetal bovine serum, 100 U/ml penicillin, and 100 μg/ml streptomycin at 37 °C in a 5% CO_2_ humidified incubator.

### Site-directed mutagenesis and generation of mutant HBV

Plasmid pT472C that carried in the HBV genome a T to C mutation at nucleotide 472 (counting from the unique EcoRI site) within the S1 target site was generated by site-directed mutagenesis of the pHBV1.3 plasmid, which carries a 1.3-fold wild-type HBV genome^44^, according to the manufacturer’s instructions (turbo pfu, Stratagene cat. no. 600250). T472C HBV was generated by hydrodynamic injection of NSG mice with 10 μg of plasmid pT472C and serum samples collected as a source for T472C HBV.

### Construction and production of shRNA-encoding plasmids and pseudotyped AAV8 vectors

The predicted shRNA sequences (Supplementary Table S1) were synthesized as duplex primer pairs and cloned into the pSuper expression plasmid (OligoEngine, Seattle, WA) and the pAAVEMBL AAV packaging plasmid as described previously^12^. The pseudotyped AAV8 vectors were produced by the triple transfection method and purified by cesium chloride sedimentation^45^.

### Co-transfection *in vitro*

Huh-7 cells in 24-well tissue culture plates (80% confluence) were co-transfected with 1 μg of shRNA- and 1 μg of wild-type HBV (pHBV1.3)- or T472C mutant HBV (pT472C)-encoding plasmids using lipofectamine 2000 (Invitrogen) according to the manufacturer’s instructions. Culture supernatants were collected three days later for analysis.

### Hydrodynamic tail vein injection

Female C57BL/6 mice (6- to 8 -weeks-old) were co-injected with 10 μg of HBV-encoding plasmid and 30 μg of shRNA-encoding pSuper plasmid in saline into the tail vein in a volume equivalent to 8% of the mouse body mass (e.g., 2 ml for a mouse of 25 g), the total volume being delivered within 5–8 seconds^46^. Mice were sacrificed three days later and serum samples collected for analysis.

### Delivery of AAV vectors

To evaluate RNAi-mediated anti-HBV effects, ICR/HBV transgenic mice and HBV-infected human liver chimeric mice were injected intravenously (i.v.) with 1 × 10^12^  vg per mouse of AAV8 vector encoding various shRNAs, as indicated in the Figure legend and serum and liver samples were collected at different times for analysis.

### Serum HBV DNA and protein analysis

Serum HBV DNA titers were quantified by a hybridization probe-based real-time polymerase chain reaction (PCR; LightCycler FastStart; Roche Diagnostics GmbH, Mannheim, Germany) as described previously^17^. Levels of hepatitis B surface antigen (HBsAg) in the culture and serum samples were measured using either a sandwich ELISA assay (General Biologicals Corp, Hsin-Chu, Taiwan) or an Elecsys HBsAg II reagent kit and a Cobas analyzer (Roche Diagnostics GmbH). Levels of hepatitis B e antigen (HBeAg) in mouse serum were measured using an Elecsys HBeAg reagent kit and a Cobas analyzer (Roche Diagnostics GmbH).

### Liver HBV DNA and RNA analysis

Total DNA and RNA were extracted from liver tissues and examined for the presence of HBV DNA and RNA by, respectively, Southern or Northern blotting as described previously^17^. The probe was generated using a random primer labeling system (Rediprime II, GE Healthcare, Piscataway, NJ) containing a ^32^P-labeled DNA fragment corresponding to the HBV small S antigen-coding sequence. Signals were visualized using a Typhoon 9410 Imager (Amersham, Bucks, UK) and analyzed using ImageQuant software (Molecular Dynamics, Sunnyvale, CA).

### HBV infection of human liver chimeric mice

*Fah*^*−/−*^*/Rag2*^*−/−*^*/Il2rg*^*−/−*^ mice were transplanted with human hepatocytes as described previously^42^. In brief, 6-week-old mice were intrasplenically transplanted with 1 × 10^6^ human hepatocytes (BD Biosciences, San Jose, CA, USA) and selected by cycling with 2-(2-nitro-4-trifluoro-methylbenzoyl)-1,3-cyclohexanedione, a drug that blocks the activity of hydroxyphenylpyruvate dioxygenase upstream of FAH in tyrosine metabolism and therefore prevents the accumulation of hepatotoxic metabolites. Human hepatocyte repopulation levels were determined by measuring human albumin levels in mouse serum using a human albumin ELISA kit (Bethyl Laboratories, Montgomery, TX, USA). Three to four months after transplantation, chimeric mice with human albumin levels greater than 1 mg/ml were subjected to HBV injection. Each animal was injected intraperitoneally with 5 × 10^7^ viral genomes of wild-type HBV, T472C HBV or a mixture of wild-type and T472C HBV at a ratio of 9:1. Wild-type HBV was obtained from ICR/HBV transgenic mice and T472C HBV from NSG mice hydrodynamically injected with pT472C plasmid.

### Immunostaining

Immunostaining of HBcAg and human FAH was performed on paraffin-embedded mouse liver tissues. Briefly, paraffin-embedded sections of fixed liver were dewaxed, rehydrated, and incubated overnight with polyclonal rabbit anti-HBcAg antibodies (Thermo Fisher Scientific, CA, USA) or polyclonal goat anti-FAH antibodies (Santa Cruz Biotechnology, Santa Cruz, CA, USA). Immunoreactivity was assessed with fluorescently labeled secondary antibodies (counterstaining with DAPI).

### Sequence analysis of the HBV genome

To analyze the development of escape mutants, the corresponding region of the HBV genome was PCR-amplified and sequenced by two methods. The first method involved direct sequencing of the PCR fragment^47^. Briefly, 10 μl of mouse serum was pretreated at 37 °C overnight with 40 units of DNase I (Roche), then viral DNA was extracted using a QuickGene DNA Tissue Kit S (Fujifilm, Tokyo, Japan). The DNA fragment covering the S1 target region of the HBV genome was then amplified by PCR using oligonucleotides (sense: 5′-CGCAGTCCCCAACCTCCAAT-3′; antisense: 5′-AAAGCCCTACGAACCA-3′), the PCR products were treated with exonuclease I (Fermentas) and shrimp alkaline phosphatase to remove the unincorporated primers and nucleotides, and the purified DNA fragments were sequenced using an automatic sequencer ABI 3730xl (Applied Biosystems, Foster City, CA) and the primer 5′-CGCAGTCCCCAACCTCCAAT-3′. The second method involved the cloning and sequencing of the individual HBV genome. Briefly, the DNA fragment covering the S1 and P28 target region of the HBV genome was amplified by PCR using oligonucleotides (sense: 5′-CGCAGTCCCCAACCTCCAAT-3′; antisense: 5′-AGAGTTATCAGTCCCGATAA-3′) and cloned into the pJET1.2 vector (CloneJET™ PCR Cloning Kit, Fermentas, Vilnius, Lithuania). The cloned HBV genome was then sequenced using pJET1.2 forward and reverse sequencing primers.

### PCR-RFLP assay

A PCR–restriction fragment length polymorphism (RFLP)-based assay was used to identify the wild-type HBV and the T472C HBV mutant in the serum^48^. The primers used for PCR amplification were: forward, 5′-TCCTGCTGCTATGCCTCATCTTCTTGTTGGTTCTTCTGGACTATCAAGGTATGTTGCCCAT-3′; reverse, 5′-GAATACAGGTGCAATTTCCG-3′. The forward primer introduced a G to A mutation at position 470 in the HBV genome, which created a BccI site (CCATCNNNN↓) in T472C HBV, but not wild-type HBV, and BccI digestion of the PCR product from T472C HBV generated two fragments of 129 and 66 bp, while the 195 bp fragment from the wild-type virus was not cut. The wild-type and T472C HBV plasmids were mixed at the indicated ratios and digested with BccI to generate different ratios of the S1-wt (195 bp) and S1-T472C (129 bp) fragments for use as a standard.

### cccDNA analysis

cccDNA was purified by a modification of alkaline lysis procedure as reported previously^49^. Briefly, the liver fragments were mechanically ground in liquid nitrogen and resuspended in lysis buffer A (150 mM NaCl, 10 mM Tris, pH 7.5, 1 mM EDTA, 0.5% NP-40) at 4 °C for 10 minutes. The cytoplasmic fraction was separated from the nuclear fraction by centrifugation at 500 g, 4 °C for 5 minutes. The pelleted nuclei were resuspended in 2 ml of Tris/EDTA (TE) buffer (10 mM Tris–HCl, pH 7.5 and 10 mM EDTA) with addition of 10 μg tRNA, then lysed by adding 2 ml of lysis buffer B (0.1 N NaOH and 6% sodium dodecyl sulfate) and incubated at 37 °C for 20 min. This step irreversibly denatured all double-stranded DNA species that were not covalently closed. After denaturation, the alkaline lysate was neutralized by adding 1 ml of 3 M potassium acetate (pH 5.2) and then centrifuged at 17,000 g, 4 °C for 20 min. Nucleic acids were purified by phenol-chloroform (1:1) and ethanol precipitation. Precipitated nucleic acid was resuspended in 0.1X TE buffer (10 mM Tris–HCl, pH 7.5 and 1 mM EDTA). For cccDNA quantification, aliquots of extracted DNA (500 ng each) were treated for 1 hour at 37 °C with 10 U plasmid safe DNase I (Epicentre Inc.). DNase was inactivated by incubating the reactions for 30 minutes at 70 °C. The amounts of intrahepatic cccDNA were determined in a Light-Cycler (Roche Diagnostics) using HBV cccDNA-specific primers and FRET hybridization probes^50^. The sequences of the forward and reverse primers were 5′-CTCCCCGTCTGTGCCTTCT-3′ and 5′-GCCCCAAAGCCACCCAAG-3′, respectively. The sequences of the FRET hybridization probes were 5′-GTTCACGGTGGTCTCCATGCGACGT-FL-3′ and 5′-R640-AGGTGAAGCGAAGTGCACACGGTCC-3′, respectively. PCR amplification was performed as follows: 95 °C for 10 minutes, then 45 cycles of 95 °C for 10 seconds, 62 °C for 10 seconds, and 72 °C for 20 seconds. Tenfold dilutions (2.66 × 10^0^ to 2.66 × 10^7^ copies/ml) of plasmid pHBV1.3 were used to generate a standard curve in parallel PCRs. The number of human hepatocytes represented in the samples was determined by SYBR-green PCR using SYBR Green reaction mix (Roche Diagnostics, Mannheim, Germany) and primers (forward: 5′-TGAGATTAGTAGTATGGGAG-3′; reverse: 5′-CACCCTATTAACCACTCACG-3′) specific for human mitochondria DNA^51^. Human genome copy numbers were determined by comparing the data of the quantitative PCR with a dilution series of purified genomic human DNA (Clontech, Mountain View, CA) under the assumption that about 6 pg genomic DNA represent one hepatocyte. To examine the frequency of escape mutants at the cccDNA level, cccDNA isolated from the different groups were used a PCR template to amplify the DNA fragment covering the S1 and P28 target region. The PCR fragment was then cloned into the pJET1.2 vector for sequencing using pJET1.2 forward and reverse sequencing primers.

### Statistical analysis

Results are presented as the mean ± standard deviation (SD). Differences between groups were examined for statistical significance using two-tailed unpaired Student’s t test; a p value < 0.05 was considered statistically significant.

## Additional Information

**How to cite this article**: Shih, Y.-M. *et al.* Combinatorial RNA Interference Therapy Prevents Selection of Pre-existing HBV Variants in Human Liver Chimeric Mice. *Sci. Rep.* **5**, 15259; doi: 10.1038/srep15259 (2015).

## Supplementary Material

Supplementary Information
