# Supplementary material for: Combinatorial RNA Interference Therapy Prevents Selection of Pre-existing HBV Variants in Human Liver Chimeric Mice
Source: Sci Rep. 2015 Oct 20;5:15259. doi: 10.1038/srep15259 (PMC4612501; doi:10.1038/srep15259)
Supplement: Supplementary Information [file srep15259-s1.pdf]

Online Supplementary Information

Combinatorial RNA Interference Therapy Prevents Selection of Pre-existing HBV Variants in Human Liver Chimeric Mice

Yao-Ming Shih[1,2], Cheng-Pu Sun[2], Hui-Hsien Chou[3], Tzu-Hui Wu[4], Chun-Chi Chen[5], Ping-Yi Wu[2], Yu-Chen Enya Chen[2], Karl-Dimiter Bissig[6] and Mi-Hua Tao[1,2*]

[1] Graduate Institute of Microbiology, National Taiwan University, Taipei, Taiwan

[2] Institute of Biomedical Sciences, Academia Sinica, Taipei, Taiwan

[3] Department of Computer Science, Iowa State University, Ames, Iowa, United States of America

[4] Department of Clinical Laboratory Sciences and Medical Biotechnology, National Taiwan University, Taipei, Taiwan

[5] CAS Key Laboratory of Pathogenic Microbiology and Immunology, Institute of Microbiology, Chinese Academy of Sciences, Beijing, China

[6] Department of Molecular and Cellular Biology, Center for Cell and Gene Therapy, Stem Cells and Regenerative Medicine Center, Baylor College of Medicine, One Baylor Plaza, BCM505, Alkek Building, N1010.07, Houston, TX 77030, USA

Supplementary Figures

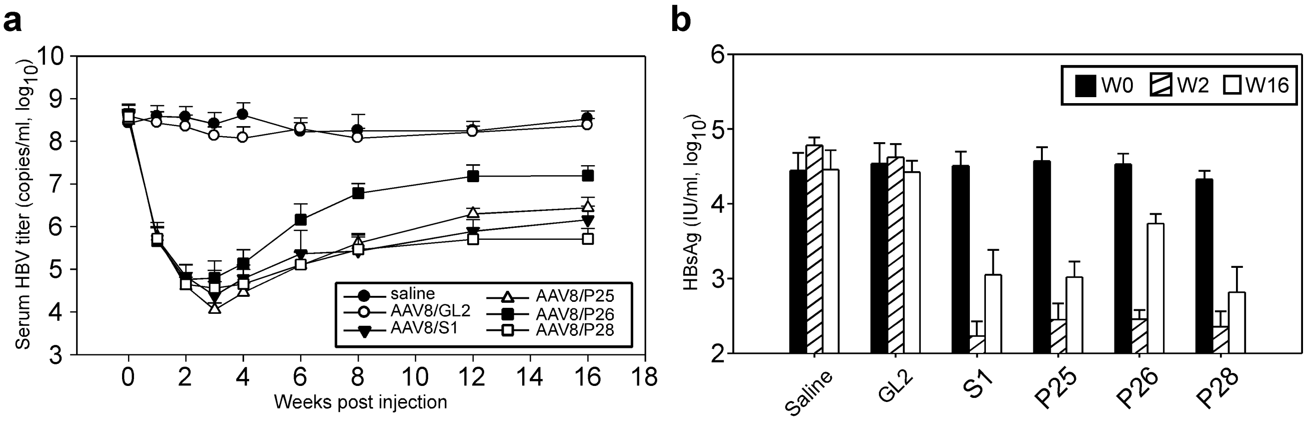

Supplementary Figure S1. Time-course of effects of AAV8/shRNA treatment in HBV transgenic mice. ICR/HBV mice (n = 7 - 9) were injected i.v. with 1×10^{12} vg per mouse of AAV8 vector encoding one of the three selected shRNAs (P25, P26, or P28), with AAV8/S1 as positive controls and AAV8/GL2 and saline groups as negative controls, and the HBV DNA titer (a) or HBsAg levels (b) measured in the serum at the indicated time point (mean ± SD).

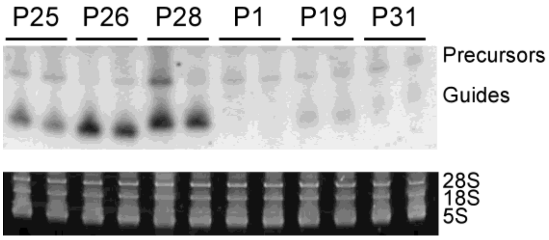

Supplementary Figure S2. Expression of small hirpin RNA in the liver. Liver samples were obtained from the AAV8/shRNA-treated HBV transgenic mice as described in the legend of Figure 3. Total liver RNA (30 μg) was analyzed by Northern blotting using specific isotope-labeled oligonucleotide probes that were complementary to the putative mature processed guides of shRNA P1, P19, P25, P26, P28 or P31 (upper panel); ethidium bromide-stained ribosomal RNA (rRNA) served as the loading controls (lower panel).

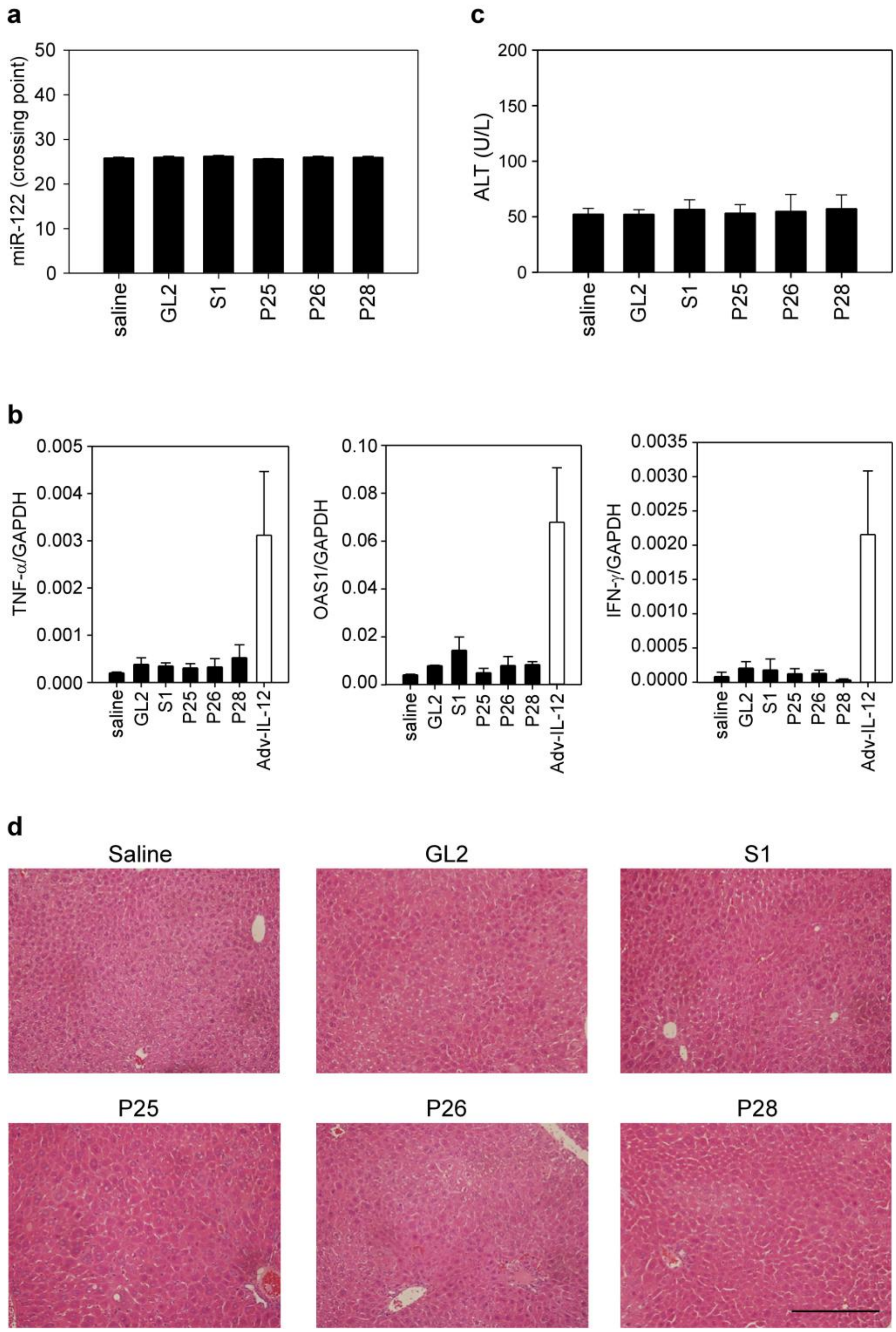

Supplementary Figure S3. Toxicity profiles of PICKY-selected shRNAs. ICR/HBV mice (n = 3 in each group) were injected i.v. with 1×10^{12} vg per mouse of AAV8 vector encoding the indicated shRNA. Mice injected with saline served as negative controls. The mice were sacrificed at week 6

post AAV injection and liver tissues collected for analysis. (a) Quantitative analysis of intracellular miR-122 by quantitative reverse transcription-PCR (RT-qPCR). Data are presented as the mean crossing point of intracellular miR-122 per µg of total RNA ± SD. (b) Total liver RNA from HBV transgenic mice was assayed for TNF-α, OAS1, and IFN-γ mRNA by RT-qPCR. Liver RNA samples from HBV transgenic mice collected 3 days after injection of 1 x 10^9 plaque forming units of adenovirus carrying the mouse IL-12 gene (Ad/IL-12) were included as controls. The results are expressed as the ratio of the RNA copy number to that for glyceraldehyde 3-phosphate dehydrogenase (GAPDH; mean ± SD). (c) Serum samples collected at 3 weeks after AAV injection were measured for the ALT activity (mean ± SD). (d) Histopathological analysis of hematoxylin- and eosin-stained liver sections of mice treated with the indicated AAV8/shRNA vectors. Original magnification, × 200; bar, 200 µm.

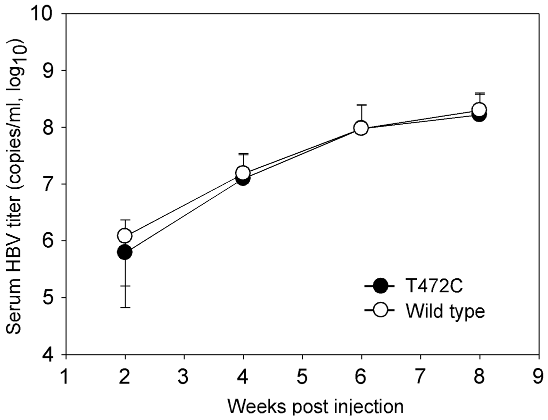

Supplementary Figure S4. Wild-type and T472C HBV infection in hu-FRG mice. Human liver chimeric mice were injected intraperitoneally with 5 x 10^7 viral genomes of wild-type or T472C HBV and the HBV DNA titer in the sera measured at the indicated time (n = 3 in each group; mean ± SD).

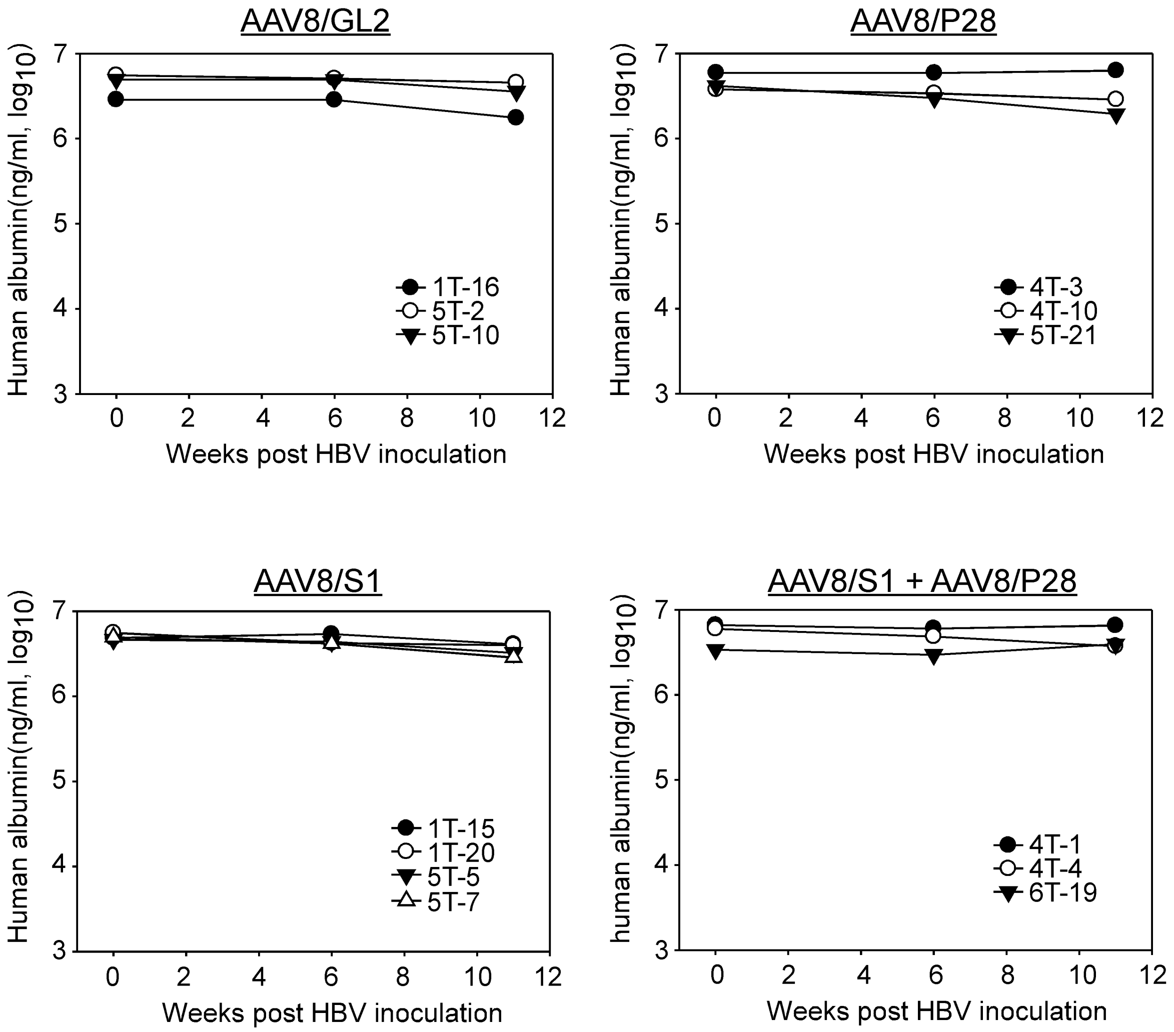

Supplementary Figure S5. Serum human albumin levels in HBV-infected hu-FRG mice treated with various AAV8/shRNA vectors. Human albumin levels in the serum from the same hu-FRG mice described in Figure 4 were determined before HBV inoculation (week 0), before AAV8/shRNA injection (week 6) and 5 weeks after AAV8/shRNA injection (week 11) by ELISA. Data are presented as means of triplicate determinations. SDs were < 10% of the mean.

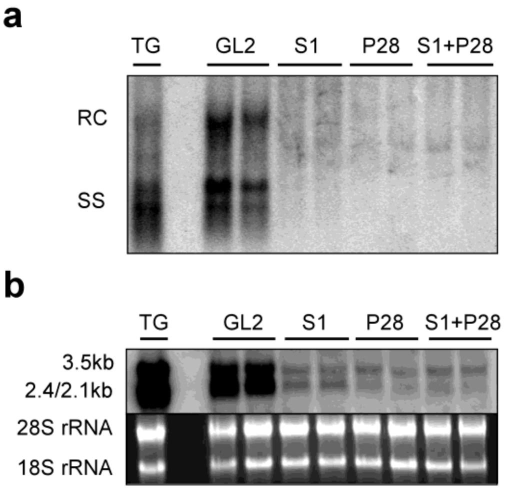

Supplementary Figure S6. Reduced intrahepatic HBV DNA and RNA levels in HBV-infected hu-FRG mice treated with the indicated AAV8/shRNA vectors. The hu-FRG mice shown in Figure 4 were sacrificed at five weeks post AAV injection and the liver tissues collected for (a) Southern and (b) Northern blot analysis. (a) Total liver DNA was analyzed for HBV replicative intermediates. Bands corresponding to the relaxed circular (RC) and single-stranded (SS) linear HBV DNA replicative forms are indicated. (b) Total liver RNA was analyzed for the 3.5 kb and 2.4/2.1 kb HBV transcripts; ethidium bromide-stained 28S and 18S ribosomal RNAs (rRNA) served as the loading controls.

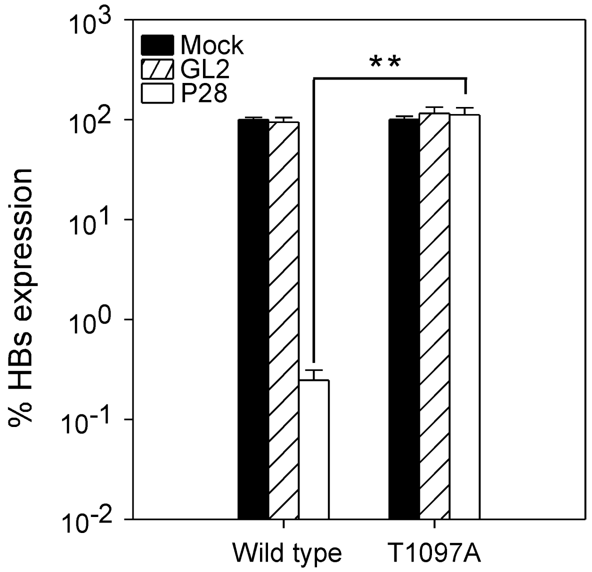

Supplementary Figure S7. A single nucleotide mutation in the siRNA target sequence resulted in loss of sensitivity to P28. C57BL/6 mice (n = 5) were hydrodynamically co-injected with shRNA- and wild-type HBV (WT, pHBV1.3) or T1097A HBV-encoding plasmids. Three days later, serum HBsAg levels were measured and are presented as a percentage of the level in mice transfected with HBV plasmid alone (mock, mean ± SD). (**P < 0.01)

Supplementary Tables

Name	shRNA target site(21nt)	HBV mRNA	Conservation between genotypes
P1	GCGCACCTCTCTTTACGCGGA	3.5, 2.4, 2.1, 0.7	B, D, F, H
P19	AGGCGGGGTTTTTCTTGTTGA	3.5, 2.4, 2.1	A, B, C, D, E, G
P20	CGGGGTTTTTCTTGTTGACAA	3.5, 2.4, 2.1	A, C, D, E, G
P21	ATGTTGCCCGTTTGTCCTCTA	3.5, 2.4, 2.1	A, B, C, D, E, F, G
P24	TTGTCTTTGGGTATACATTTA	3.5, 2.4, 2.1	A, B, C, D, E, G
P25	TGGGTATACATTTAAACCCTA	3.5, 2.4, 2.1	A, C, D
P26	GGGTATACATTTAAACCCTAA	3.5, 2.4, 2.1	A, C, D
P28	CAGGCTTTCACTTTCTCGCCA	3.5, 2.4, 2.1	A, C, E
P29	AGGCTTTCACTTTCTCGCCAA	3.5, 2.4, 2.1	A, C, E
P31	CTGCCGATCCATACTGCGGAA	3.5, 2.4, 2.1	A~H

Supplementary Table S1. Sequence candidates selected by PICKY software

Oligonucleotides	Sequence (5’-3’)
P1 sense	5'-GAAATTAATACGACTCACTATAGGGCGCACCTCTCTTTACGCGGA-3'
P1 antisense	5'-TCCGCGTAAAGAGAGGTGCGCCCTATAGTGAGTCGTATTAATTTC-3'
P19 sense	5'-GAAATTAATACGACTCACTATAGGAGGCGGGGTTTTTCTTGTTGA-3'
P19 antisense	5'-TCAACAAGAAAAACCCCGCCTCCTATAGTGAGTCGTATTAATTTC-3'
P25 sense	5'-GAAATTAATACGACTCACTATAGGTGGGTATACATTTAAACCCTA-3'
P25 antisense	5'-TAGGGTTTAAATGTATACCCACCTATAGTGAGTCGTATTAATTTC-3'
P26 sense	5'-GAAATTAATACGACTCACTATAGGGGGTATACATTTAAACCCTAA-3'
P26 antisense	5'-TTAGGGTTTAAATGTATACCCCCTATAGTGAGTCGTATTAATTTC-3'
P28 sense	5'-GAAATTAATACGACTCACTATAGGCAGGCTTTCACTTTCTCGCCA-3'
P28 antisense	5'-TGGCGAGAAAGTGAAAGCCTGCCTATAGTGAGTCGTATTAATTTC-3'
P31 sense	5'-GAAATTAATACGACTCACTATAGGCTGCCGATCCATACTGCGGAA-3'
P31 antisense	5'-TTCCGCAGTATGGATCGGCAGCCTATAGTGAGTCGTATTAATTTC-3'

Supplementary Table S2. Sequences of oligonucleotide pairs used to generate DNA templates for synthesis of small RNA probes by *in vitro* transcription are shown as follows (with the mature shRNA sequence underlined)

Supplementary Materials and Methods

Small RNA northern. For small RNA northern blot analysis, 20 µg of total liver RNA from AAV8/shRNA-treated animals was separated on a 15% polyacrylamide–urea gel, transferred to a Hybond-N+ membrane (GE Healthcare, Chalfont, St. Giles, UK), and hybridized to ^{32}P-labeled short RNA probe. Short RNA probes complementary to each of the shRNAs were prepared by *in vitro* transcription using T7 RNA polymerase as described previously[1]. The sequences of oligonucleotide pairs used to generate DNA templates for T7 RNA polymerase are shown in Supplementary Table S2. Radioactive signals were visualized using a Typhoon 9410 Imager (Amersham, Bucks, UK) and analyzed using ImageQuant software (Molecular Dynamics, Sunnyvale, CA).

Measurement of serum alanine aminotransferase activity. Serum alanine aminotransferase (ALT) activity was measured using Vitros Chemistry Products ALT slides and a Vitros 950 chemical analyzer (Johnson & Johnson, Rochester, NY). Values are expressed as units per liter (U /L).

Quantification of miR-122. miR-122 levels were determined by stem-loop RT-qPCR as described previously[2]. The sequences of the oligonucleotides used were: retrotranscription stem-loop oligo, 5'-GTTGGCTCTGGTGCAGGGTCCGAGGTATTCGCACCAGAGCCAACCAAACA-3'; PCR forward primer, 5'-TTCCGTGGAGTGTGACAATGG-3' ; and PCR reverse primer, 5'-GTGCAGGGTCCGAGGT-3'.

Histology. Liver sections were fixed in 4% paraformaldehyde in phosphate-buffered saline, embedded in paraffin, sectioned (5 μm), and stained with hematoxylin and eosin, and then were mounted and observed by light microscopy.

Expression of cytokine and cytokine-induced genes. One microgram of total liver RNA was reverse transcribed (Roche, Mannheim, Germany) and subjected to quantitative real-time PCR (FastStart SYBR Green Master; Roche Diagnostics GmbH) using specific primer sets as reported previously[3]. All samples were run in duplicate, together with a negative control with no reverse transcriptase and water blanks. mRNA levels were assessed from the cycle number at which the cytokine or chemokine amplification exceeded the threshold crossing point (ct) and these values were standardized against the GAPDH mRNA value for the same sample.

Supplementary References

1 Chu, Y. D. *et al.* RACK-1 regulates let-7 microRNA expression and terminal cell differentiation in Caenorhabditis elegans. *Cell Cycle* **13**, 1995-2009 (2014).

2 Varkonyi-Gasic, E., Wu, R., Wood, M., Walton, E. F. & Hellens, R. P. Protocol: a highly sensitive RT-PCR method for detection and quantification of microRNAs. *Plant Methods* **3**, 12 (2007).

3 Chen, C. C. *et al.* Long-term inhibition of hepatitis B virus in transgenic mice by double-stranded adeno-associated virus 8-delivered short hairpin RNA. *Gene Ther* **14**, 11-19 (2007).
